# Family risk factors, dyadic coping, and family resilience in young stroke dyads: an actor-partner interdependence mediation model

**DOI:** 10.3389/fpsyt.2026.1826436

**Published:** 2026-06-05

**Authors:** Lili Zhu, Hui Han, Huijuan Wang, Jiaru Xie, Xue Cheng, Wanyin Song, Lei Huang, Hua Zhang

**Affiliations:** 1 School of Nursing, Xinxiang Medical University, Xinxiang, China; 2 Department of Nursing, The First Affiliated Hospital of Xinxiang Medical University, Xinxiang, China; 3 Department of Nursing, The Third Affiliated Hospital of Xinxiang Medical University, Xinxiang, China

**Keywords:** actor–partner interdependence mediation model, couple, dyadic coping, family resilience, stroke, young adults

## Abstract

**Objective:**

Stroke in young individuals constitutes a significant familial stressor, with ensuing family risk factors critically influencing recovery. While couples’ collaborative coping strategies play a vital role in enhancing family resilience, the mechanisms underlying this process remain largely unexplored. This study aims to elucidate the impact of familial risk factors and dyadic coping on the family resilience of young stroke.

**Methods:**

This cross-sectional study integrates evidence-based research with clinical data. We developed a structured questionnaire grounded in identified familial risk factors. A cohort of 243 dyads, comprising young stroke patients and their spouses, was recruited from the neurology departments of four tertiary hospitals. The Actor–Partner Interdependence Mediation Model (APIMeM) framework was utilized, in which patients were treated as actors (influencing their own outcomes) and spouses as partners (influencing each other’s outcomes), to determine both direct and indirect effects among variables, with significance evaluated using bootstrap procedures (5,000 resamples, 95% confidence intervals). Model simplification was guided by chi-square difference testing.

**Results:**

The analysis indicated that family resilience could be strengthened in young stroke survivors and their spouses. APIMeM analyses showed that patients’ and spouses’ anxiety (*β* = –0.479, *P* = 0.002) and depression (*β* = –0.718, *P* < 0.001) had significant negative actor effects on their own family resilience, while patients’ activities of daily living (ADL) had significant positive actor *(β = 0.175, P* < 0.001) and partner effects (*β* = 0.198, *P* < 0.001) on family resilience. Crucially, dyadic coping partially mediated these relationships, with significant actor-actor indirect effects for anxiety (*β* =–0.139, 95% *CI* [–0.261, –0.029]), depression (*β* = –0.190, 95% *CI* [–0.321, –0.084]), and ADL (*β* = 0.062, 95% *CI* [0.009, 0.131]) on family resilience. Significant actor-partner mediation effects were also observed for depression (*β* = –0.072, 95% *CI* [–0.170, –0.017]) and ADL (*β* = 0.028, 95% *CI* [0.002, 0.079]).

**Conclusions:**

Using a dyadic APIMeM approach, this study demonstrates that dyadic coping serves as a key mediator linking familial risk factors to family resilience in young stroke dyads. Resilience emerges as a shared, co-constructed process rather than an individual attribute, underscoring the need for interventions targeting the couple as a unit to mitigate intervention bias.

## 1 Introduction

Stroke is a sudden cerebrovascular event and one of the leading causes of death and disability among adults (1). While stroke rates are declining among adults over 75, there is an alarming increase among younger adults, primarily due to uncontrolled risk factors such as obesity and hypertension (2, 3). This trend has turned stroke among young adults into a significant public health concern, particularly in high-risk regions like Iran, Libya, Nigeria, Chile, the United States, China, and Australia (4).

Young adults who experience a stroke often face abrupt disruptions in their careers, financial instability, and shifts in social roles and responsibilities (5, 6). These challenges place unique psychosocial stressors on families, differing significantly from older populations with more established life routines and roles. Understanding how these families cultivate resilience in the face of adversity is crucial but remains under-explored in current research.

Family resilience, defined as the ability of a family to recover and adapt following difficulties and setbacks (7), plays a critical role in patient and spouse health outcomes. Diminished family resilience is associated with increased levels of post-traumatic stress disorder (PTSD) among patients and their spouses (8). Conversely, high levels of family resilience enhance family quality of life, reduce readmission rates, and mitigate family crises (9, 10). However, few studies have specifically examined the underlying mechanisms of family resilience in young stroke patients.

Family resilience plays a crucial role in how families adapt to and overcome the challenges posed by stroke. The Double ABCX Model, introduced by McCubbin and Patterson (11), also known as the Family Resilience Model, builds upon family stress theory and family systems theory. This model suggests that when a family encounters stress, it triggers a crisis that prompts the mobilization of resources for coping. Successful coping enhances the family’s adaptation and balance, leading to improved outcomes. For example, family members may develop a better understanding of the disease’s impact, no longer perceive stroke as terrifying, and distance themselves from negative emotions (12). Patients and caregivers may believe they can manage recovery tasks and feel confident about the future.

However, not all families are equally prepared to handle adversity. Recognizing this, Patterson (13) proposed that family resilience emerges from processes of struggle and development, involving both family risk factors and protective factors. Understanding these factors is essential to enhancing family resilience among stroke patients, particularly in young adults.

To identify the factors that protect or impede family resilience among stroke patients, our research group conducted a systematic review and meta-analysis. We systematically searched the literature using subject headings and keywords related to family resilience and stroke. The review identified key factors influencing family resilience at the individual, familial, and societal levels. Among these factors, family risk predominantly involves negative stress reactions and the health status of both patients and caregivers. Depression and anxiety each affect approximately one in three stroke patients during the first year after a stroke (14). Similarly, the prevalence of depressive symptoms was 40.2%, and that of anxiety symptoms was 21.4% among the main caregivers of stroke patients (15). The activities of daily living (ADL) in stroke patients are crucial indicators of their physical condition. ADL assessments evaluate a patient’s ability to perform basic self-care tasks, such as bathing, dressing, eating, toileting, and mobility (16). Based on this evidence, we identified the patient’s ADL, as well as the anxiety and depression of both the patient and their spouse, as critical family risk factors for this study.

In this study, the term “family risk factors” is used to denote individual-level psychological symptoms (anxiety, depression) and functional status (ADL) of both the patient and the spouse, which, according to the Double ABCX Model (11), act as primary or secondary family stressors. Although measured at an individual level, these factors emerge from the shared familial context of stroke and have profound, interdependent effects on the overall family system’s adaptive capacity. Therefore, they are conceptualized as critical family-level risk constructs that threaten family resilience.

Stroke is a stressful family event that affects both partners, impacting their emotional and intimate relationship (17). During the recovery period, both partners need to adapt to changes in individual roles, family dynamics, and interpersonal relationships. Studies have shown that when one partner faces a stressor, the other partner’s supportive behaviors—such as expressing empathy, providing support, active listening, or sometimes avoidance—are activated. This process is known as dyadic coping (18). Research by Pucciarelli (19) has demonstrated that active dyadic coping between stroke patients and their spouses can enhance family quality of life, alleviate negative emotions like anxiety and depression, and promote family harmony and recovery. However, the specific role of dyadic coping in the family resilience of young stroke couples remains unclear.

Examining family resilience solely from an individual perspective may introduce bias, as it overlooks the dynamics of the family unit as a whole (20). To address this gap, this study adopts a family-centered perspective to investigate the resilience of young stroke couples, emphasizing their shared experiences and mutual adaptation. Dyadic coping represents a dynamic, transactional process in which both partners’ stress experiences and coping mechanisms are interdependent. The Actor-Partner Interdependence Model (APIM) posits that interactions between individuals in a close relationship affect both their own and their partner’s outcomes (21). According to this model, patient-related factors influence not only their own family resilience but also that of their spouse.

Building upon this framework, in this study, we propose that dyadic coping mediates the relationship between family risk factors and family resilience in young stroke couples. By employing the Actor-Partner Interdependence Mediation Model (APIMeM) (22), we aim to verify the dyadic mediation effects of dyadic coping between family risk factors and family resilience in this population. This model allows us to confirm the actor effects on individuals’ own outcomes and the partner effects on their spouses, thereby uncovering the underlying mechanisms within paired relationships.

Within the Double ABCX framework, the accumulation of stressors (aA factor)—represented here by both partners’ anxiety and depression (secondary stressors) and the patient’s limited ADL (a primary stressor)—can deplete family resources and render adaptation more difficult. This study posits that dyadic coping, conceptualized as a key family resource and transactional process (bC factor), is a critical mediator on the path from these stressors to family resilience (adaptation, xX factor). Thus, the APIMeM is employed to empirically test this theoretical pathway, examining how dyadic coping transmits the actor and partner effects of family risk factors on family resilience.

### 1.1 Study hypotheses

Based on all the above literature evidence, we proposed the following research hypotheses:

1. Family risk factors (anxiety, depression, and ADL) will affect their own family resilience and influence their partners’ family resilience;
2. Dyadic coping will affect their own family resilience and influence their partners’ family resilience;
3. Dyadic coping will mediate the actor and partner effects of anxiety, depression, and ADL on family resilience.

## 2 Methods

### 2.1 Study design

This was a cross-sectional study with a prospective data collection component. This study adhered to the STROBE (Strengthening the Reporting of Observational Studies in Epidemiology) guidelines for cross-sectional studies.

### 2.2 Participants

Between February 2022 and September 2022, we recruited young stroke patients and their spouses from the neurology departments of four tertiary hospitals in Zhengzhou, Xinxiang, Luohe, and Anyang, China. Prior to discharge, patient-spouse pairs were invited to participate in the study. A simple random sampling method was used to select participants, ensuring that each patient and spouse had an equal chance of being included in the study. Specifically, at each of the four participating hospitals, research assistants screened all consecutive admissions to the neurology departments. A daily list of patient-spouse pairs who met the inclusion criteria and were scheduled for discharge within the next 48 hours formed the daily sampling frame. From this frame, dyads were selected using a computer-generated random number sequence and were then invited to participate. The inclusion criteria were (1): patients diagnosed with ischemic or hemorrhagic stroke according to the diagnostic criteria of the Chinese Medical Association Neurology Branch and confirmed by radiographic evidence; (2) patients who had passed the acute phase of stroke (more than two weeks post-onset); (3) patients able to communicate effectively without hearing impairments; (4) patients aged between 18 and 59 years; (5) Spouses aged 20 years or older (according to *The Civil Code of the People’s Republic of China*, the legal minimum age for marriage is 20 years for women and 22 years for men) and who had been living with the patient for at least one year (23); (6) Both patients and spouses willing to participate in the survey and who provided written informed consent.

Families were excluded if: (1) the family had experienced other significant traumatic events within the past year; (2) the patient had been diagnosed with a transient ischemic attack, serious organ dysfunction, or malignant tumor.

### 2.3 Sample size

According to other studies, young and middle-aged stroke patients’ score of family resilience was 190.28 ± 22.15, 95% *CI* (187.13, 193.43). Allowable error is half of the confidence interval equal to 3.15 (24).


$$n=\frac{u_{1-\alpha /2}^{2}S^{2}}{d^{2}}n=\frac{1.96^{2}\times 22.15^{2}}{3.15^{2}}=198.94\approx 199$$

Considering the questionnaire has a 15% non-response rate, it is necessary to include a minimum of 235 cases (199 ÷ 0.85≈235). Because this study is a couple of young and middle-aged stroke patients, it will require a minimum of 235 patient-spouse dyads.

### 2.4 Data collection

Before discharge, trained researchers screened stroke patients and spouses for eligibility. After obtaining signed informed consent, patient–spouse pairs completed paper-based questionnaires simultaneously in separate quiet areas. Couples were instructed not to discuss answers until both had finished, thereby minimizing response contamination.

### 2.5 Measures

#### 2.5.1 Demographics characteristics

Self-designed general information questionnaire, including patient information such as age, gender, educational level, spouse information such as age, gender, working status, and family information such as living district and monthly income.

#### 2.5.2 Family resilience assessment scale

Dr. Yan Dai developed this scale in 2008, which combined family resilience theories with local Chinese culture (25). It is a 49-item scale composed of ten dimensions and two subscales (i.e. family beliefs and family strength). The scale was based on a Likert 5-point scale. Scores range from 49 to 245, with a theoretical median of 147 for the scale. A higher total score indicated greater family resilience. The measure was initially applied to adolescents and was widely used in chronic diseases. Researchers collected data through patients and spouses, separately. In the present study, Cronbach’s α was 0.917 among the patients and 0.909 among the spouses.

#### 2.5.3 Chinese version of the dyadic coping inventory

DCI, developed by Gmelch et al. in 2008 (26), assesses stress coping in patients and their spouses during the disease process. In this study, we used the Chinese version, C-DCI, developed by Chinese scholars Xu et al. in 2016 (27). The scale consists of six subscales, a total of 37 items: common dyadic coping, negative dyadic coping, stress communication, supportive dyadic coping, delegated dyadic coping, and coping evaluation. Respondents rated each item on a 5-point Likert scale. Higher scores reflect a more positive dyadic coping style. A total score below 111 was categorized as low, while scores ranging from 111 to 145 were considered medium. Scores exceeding 145 were classified as high. In our study, Cronbach’s α was 0.944 for patients and 0.945 for spouses.

#### 2.5.4 Hospital anxiety and depression scale

The Hospital Anxiety and Depression Scale (HADS) (28) was utilized by researchers. This scale is commonly used in China (29) and comprises two subscales, each with 7 items. When the subscale scores for anxiety and depression are ≥8, it indicates symptoms of anxiety and depression. The subscale’s total score ranges from 0 to 21, with higher scores indicating more severe depression symptoms. In our study, Cronbach’s α was 0.811 for patients and 0.815 for spouses.

#### 2.5.5 Chinese version of Modified Barthel Index

To assess participants’ activities of daily living (ADL), researchers evaluated their functional ability using the MBI. The Chinese version of MBI was published by Leung and Shah in 2007 (30). The MBI score ranges from 0 (complete dependence in ADL) to 100 points (complete independence in ADL). This scale comprises ten assessment items, which include bowel management, bladder management, feeding, bathing, grooming, clothing, toilet use, transfer, walking, and climbing stairs (31).

### 2.6 Ethical statement

Ethical approval was provided by the *Ethical Approval Board* (Ethical approval code: 20220215).

### 2.7 Data analysis

**Figure 1:**
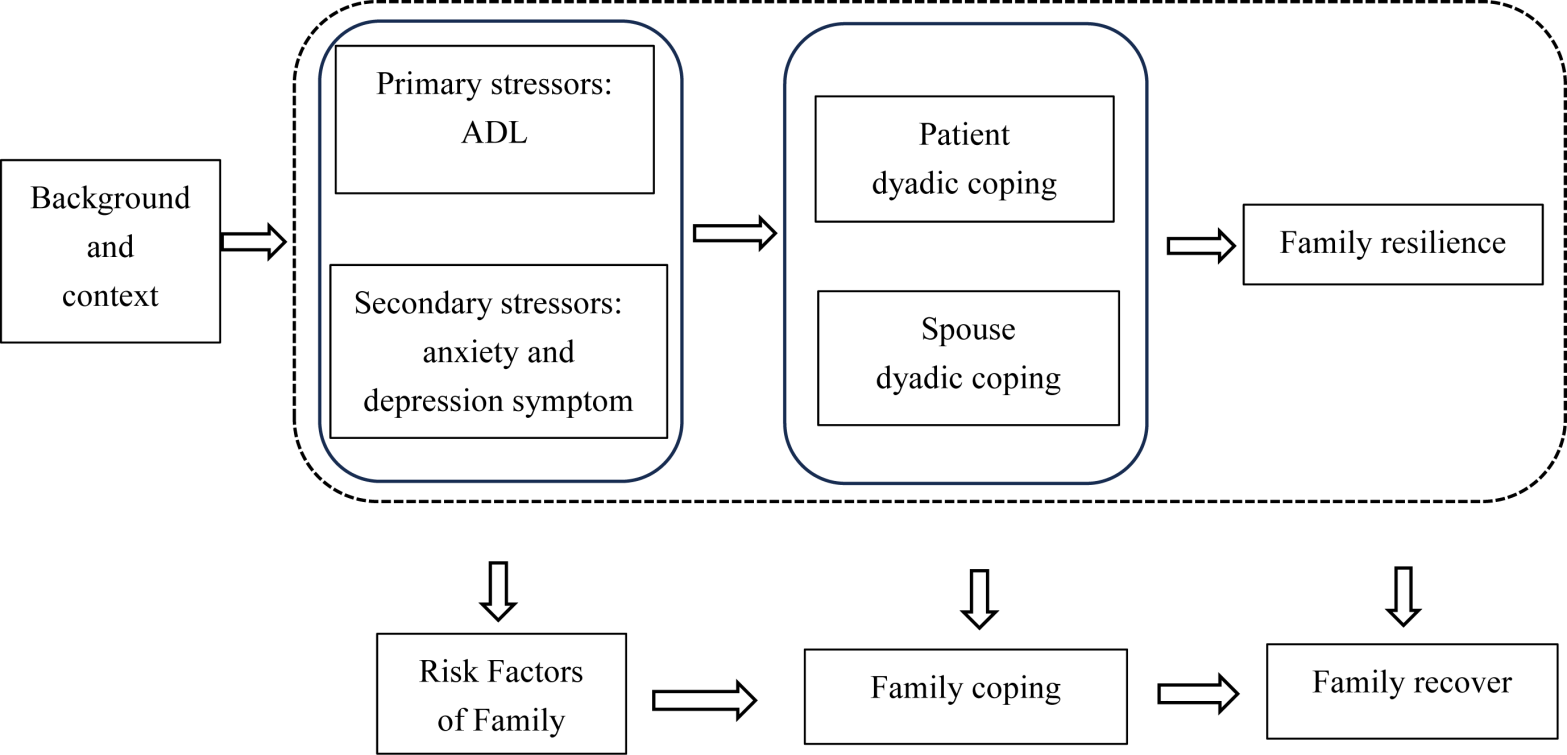
This diagram represents the framework of the study.

This study used SPSS 25.0 and AMOS 24.0 for statistical analysis. Descriptive data were presented as means and standard deviations (SD). Pearson correlation was applied to assess relationships between dyad variables. The APIMeM approach examined how anxiety, depression, and dyadic coping affected their own (actor effect) and their partners’ family resilience (partner effect) in young stroke survivors and their spouses. Mediation analysis included two independent variables (family risk factors for patients and spouses), two outcome variables (family resilience for patients and spouses), and two mediator variables (dyadic coping for patients and spouses). Researchers determined the significance of direct and indirect effects with a bootstrap sample of 5000 and a 95% confidence interval (CI) (21). The study framework is presented in **Figure 1**.

In studies of heterosexual couples, gender often plays a crucial role. Researchers commonly build a distinguishable APIMeM standard model to account for gender differences. A previous study noted that distinguishing the standard model is more complex. When *P* > 0.20, no significant difference in goodness of fit was found between the standard and simplified models (22). Following the protocol, researchers simplified the standard model by constraining each effect to be equal across the two groups and tested the models independently.

## 3 Results

### 3.1 Demographic characteristics of the dyads

The study collected 560 questionnaires, evenly distributed between 280 patients and 280 spouses. Of these, 243 couples valid responses were obtained, resulting in an 86.79% valid response rate. The remaining 37 questionnaires were invalid due to incomplete data from either the patient or the spouse.

The mean age of patients was 53.37 ± 5.695 years, while the mean age of spouses was 53.2 ± 6.258 years. The majority of patients (93.42%) and spouses (91.36%) were aged 45–59 years, positioning these dyads at a life stage of peak family and economic responsibilities. 69.55% of patients were male, and 69.55% of spouses were female, reflecting the traditional role of women as primary caregivers in Chinese society. Clinically, ischemic stroke was the predominant type (86.83%). The majority (62.96%) were first-ever strokes, yet 37.04% had experienced recurrent strokes. Nearly half of patients (48.56%) had moderate-to-severe functional impairment (dysfunction score ≥ 2). In terms of socioeconomic status, 48.97% of families reported a monthly per capita income below 3,000 *CNY*, and 47.33% resided in rural villages. Additionally, the majority of participants had completed junior high school education or below (68.31% of patients, 77.37% of spouses). **Table 1** summarizes the demographic characteristics of the dyads. A t-test revealed that stroke type, first-stroke occurrence, and the degree of functional impairment significantly influenced family resilience for both patients and spouses (*P* < 0.05) (See **Supplementary File 1**).

**Table 1:** Characteristics of the dyads and current status of scores for variables (N=243).

Characteristic/variables		Stroke survivors	Spouses
		Means (SD) or n (%)	Means (SD) or n (%)
Sociodemographic information			
Gender	Man	169 (69.55)	74 (30.45)
	Woman	74 (30.45)	169 (69.55)
Age	<45	16 (6.58)	21 (8.64)
	45-59	227 (93.42)	222 (91.36)
Educational background	Primary school and below	63 (25.93)	85 (34.98)
	Junior high school	103 (42.39)	103 (42.39)
	High school or polytechnic school	54 (22.22)	40 (16.46)
	Junior college	18 (7.41)	11 (4.53)
	Bachelor degree or above	5 (2.06)	4 (1.65)
Occupational status	Employed	89 (36.63)	84 (34.57)
	Self-employed	143 (58.58)	138 (56.79)
	Unemployed	11 (4.53)	21 (8.64)
Clinical information			
Type of stroke	Ischemic stroke	211 (86.83)	
	Hemorrhagic stroke	27 (11.11)	
	Mixed type	5 (2.06)	
Comorbid states	0-1	86 (35.39)	
	2-3	134 (55.14)	
	4-6	23 (9.47)	
First stroke	Yes	153 (62.96)	
	No	90 (37.04)	
Dysfunction	0-1	125 (51.44)	
	2-3	109 (44.86)	
	4-6	9 (3.70)	
Family information			
Monthly per capita income (CNY)	< 3000	119 (48.97)	
	3000-5000	105 (43.21)	
	>5000	19 (7.82)	
Permanent home address	Village	115 (47.33)	
	Counties	25 (10.29)	
	City	103 (42.39)	
Family resilience		179.90 ± 19.24	181.34 ± 17.53
Dyadic coping		113.20 ± 18.35	114.16 ± 18.35
Anxiety		10.93 ± 4.64	9.30 ± 3.76
Depression		10.91 ± 5.01	9.08 ± 3.74
ADL		79.60 ± 24.31	

The exchange rate in 2022 was US$1.00≈6.7261CNY.

CNY, Chinese Yuan.

### 3.2 Current scores for anxiety symptoms, depression symptoms, ADL, dyadic coping, and family resilience

In this study, the average family resilience score for young stroke patients was 3.67 ± 0.39, with 48.15% scoring below this average. For spouses, the average score was 3.70 ± 0.36, with 39.92% scoring below their average (See **Table 1**). The results showed that spouses had significantly higher family resilience scores than patients (*P* < 0.05). Additionally, young stroke survivors exhibited significantly higher levels of anxiety and depression compared to their spouses (*P* < 0.05) (See **Supplementary File 2**).

### 3.3 Anxiety symptom, depression symptom, ADL, dyadic coping, and family resilience−correlations between dyad members

**Table 2** displays dyadic correlations. Patients’ and spouses’ family resilience were positively correlated with their own dyadic coping and the patient’s ADL, and negatively correlated with their own depression. Significant cross-partner correlations were also observed.

**Table 2:** Anxiety symptom, depression symptom, ADL, dyadic coping, and family resilience−correlations between dyad members (N = 243).

		1	4	5	6	7	1	2	3	4
patient	1.family resilience	1								
	2.anxiety	-0.273**	1							
	3.depression	-0.398**	0.738**	1						
	4.ADL	0.409**	-0.299**	-0.457**	1					
	5.dyadic coping	0.539**	-0.211**	-0.268**	0.183**	1				
spouse	1.family resilience	0.813**	-0.185**	-0.294**	0.425**	0.499**	1			
	2.anxiety	-0.063	0.549**	0.292**	-0.115	-0.024	-0.099	1		
	3.depression	-0.195**	0.468**	0.542**	-0.297**	-0.125	-0.245**	0.596**	1	
	4.dyadic coping	0.475**	-0.198**	-0.250**	0.147*	0.888**	0.484**	-0.051	-0.139*	1

*P<0.05; **P<0.01.

### 3.4 Mediation analysis with APIMeM

Although Pearson correlation analysis did not reveal a statistically significant relationship between spouses’ anxiety and the family resilience of patients and their spouses. However, Hayes (32) suggested that the absence of a direct effect does not preclude an indirect effect. Therefore, the researchers chose to retain spouse anxiety as an independent variable.

Researchers included demographic factors as covariates in the model only if they demonstrated statistical significance in the univariate analysis. Although no significant differential impact of gender on family resilience was identified, evidence suggests that families with female patients and caregivers generally face worse outcomes. This is likely due to increased task difficulty and higher rates of depression among females (33, 34). Consequently, in the path analysis, the model accounted for potential confounding variables such as type of stroke, first-time occurrence, degree of functional impairment, and gender.

#### 3.4.1 Anxiety symptoms, dyadic coping, and family resilience in stroke dyads

**Figure 2:**
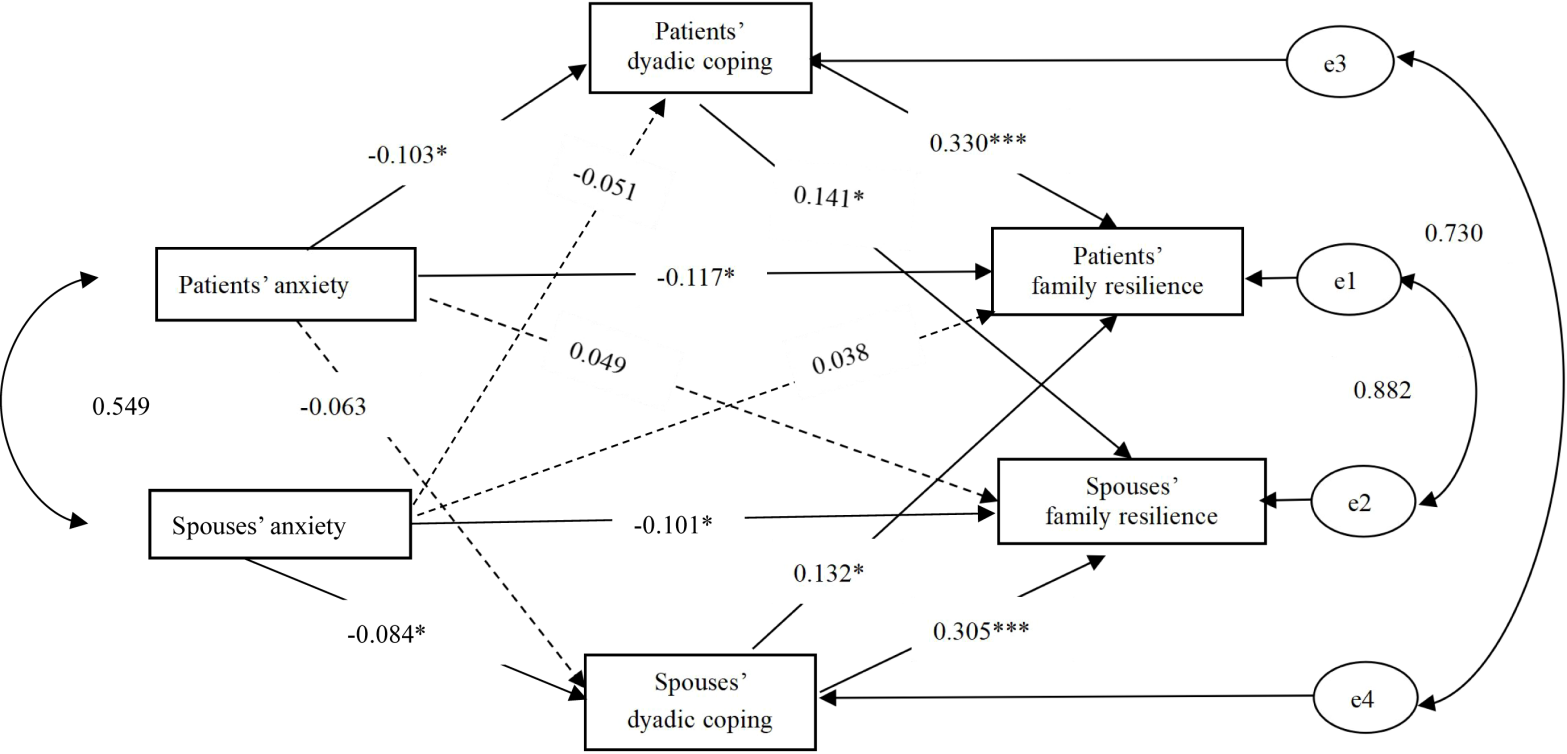
APIMeM results of anxiety symptom and dyadic coping on family resilience. In the diagram, dotted lines represent non-statistically significant path coefficients, while solid lines indicate statistically significant path coefficients. All path estimates shown in the figure are standardized. Demographic variables were controlled for in the analysis. *P< 0.05, ***P < 0.001.

The hypothesized APIMeM (**Figure 2**) showed an excellent fit (*χ*²/df = 1.397, RMSEA = 0.040, AGFI = 0.938, GFI = 0.993, NFI = 0.991, CFI = 0.997, TLI = 0.980). A simplified model with constrained actor and partner effects was adopted (*χ*²=8.379, *P* = 0.212).

Anxiety demonstrated significant negative actor effects on both individuals’ own dyadic coping (*β* = -0.407, *P* = 0.016) and family resilience (*β* = -0.479, *P* = 0.002). No significant partner effects were found. Dyadic coping significantly predicted both the actor’s (*β* = 0.341, *P* < 0.001) and partner’s (*β* = 0.137, *P* = 0.004) family resilience.

Mediation analyses (**Table 3**) revealed that individuals’ dyadic coping partially mediated the actor effect of anxiety on family resilience (*β* = -0.139, 95%*CI* = -0.261~-0.029) and fully mediated the partner effect (*β* = -0.056, 95%*CI* = -0.056~-0.009).

**Table 3:** Total effects, indirect effects, and direct effects of patients’ and spouses’ anxiety on family resilience via dyadic coping in the APIMeM.

		Unstandardized estimate	Lower	Upper	*P*
Actor effect (Individual’s anxiety →Individual’s FR)					
Total effects		-0.653	-0.983	-0.321	<0.001
Total IE		-0.173	-0.322	-0.038	0.013
**Actor-actor simple IE**	**Individual anxiety→ individual DCI→ individual FR**	**-0.139**	**-0.261**	**-0.029**	**0.013**
Partner-partner simple IE	Individual anxiety→ partner DCI→ individual FR	-0.034	-0.104	0.002	0.069
**Direct effect**	**Individual anxiety→ individual FR**	**-0.479**	**-0.783**	**-0.176**	**0.001**
Partner effect (Individual’s anxiety →Partner’s FR)					
Total effects		0.049	-0.256	0.394	0.708
Total IE		-0.141	-0.291	-0.007	0.038
**Actor-partner simple IE**	**Individual anxiety→ individual DCI→ partner FR**	**-0.056**	**-0.145**	**-0.009**	**0.010**
Partner- actor simple IE	Individual anxiety→ partner DCI→ partner FR	-0.085	-0.199	0.019	0.103
**Direct effect**	**Individual anxiety→ partner FR**	**0.189**	**-0.090**	**0.498**	**0.189**

The bold values represent significant paths (P < 0.05, with the confidence interval not including 0).

#### 3.4.2 Depression symptoms, dyadic coping, and family resilience in stroke dyads

**Figure 3:**
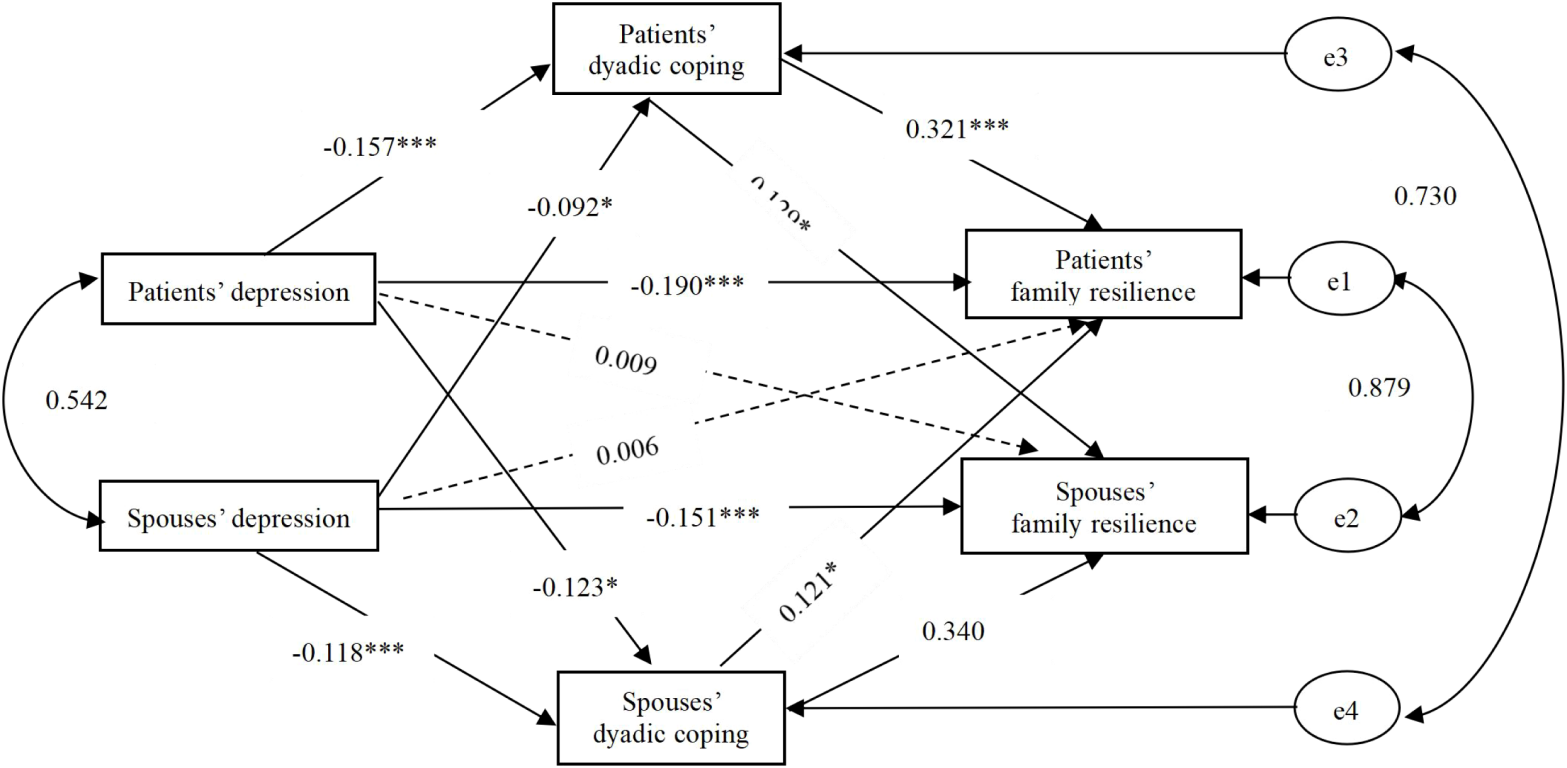
APIMeM results for the effects of depression symptoms and dyadic coping on family resilience. In **Figures 3**, dotted lines indicate non-significant path coefficients, and solid lines indicate statistically significant path coefficients. All path estimates are standardized. Demographic variables were included as covariates in the analysis. *P< 0.05, ***P < 0.001.

The hypothesized model (see **Figure 3**) provided a good fit to the data: *χ*²/df =1.024, RMSEA = 0.010, AGFI = 0.954, GFI = 0.995, NFI = 0.994, CFI = 1.000, TLI = 0.999. We found no statistically significant difference between the model with imposed constraints and the unconstrained model (*χ*²=6.143, *P* = 0.407, *P>*0.2). Therefore, we adopted a simplified model in which the actor effects of patients and spouses were constrained to be equal, and the partner effects of patients and spouses were also constrained to be equal.

**Figure 4:**
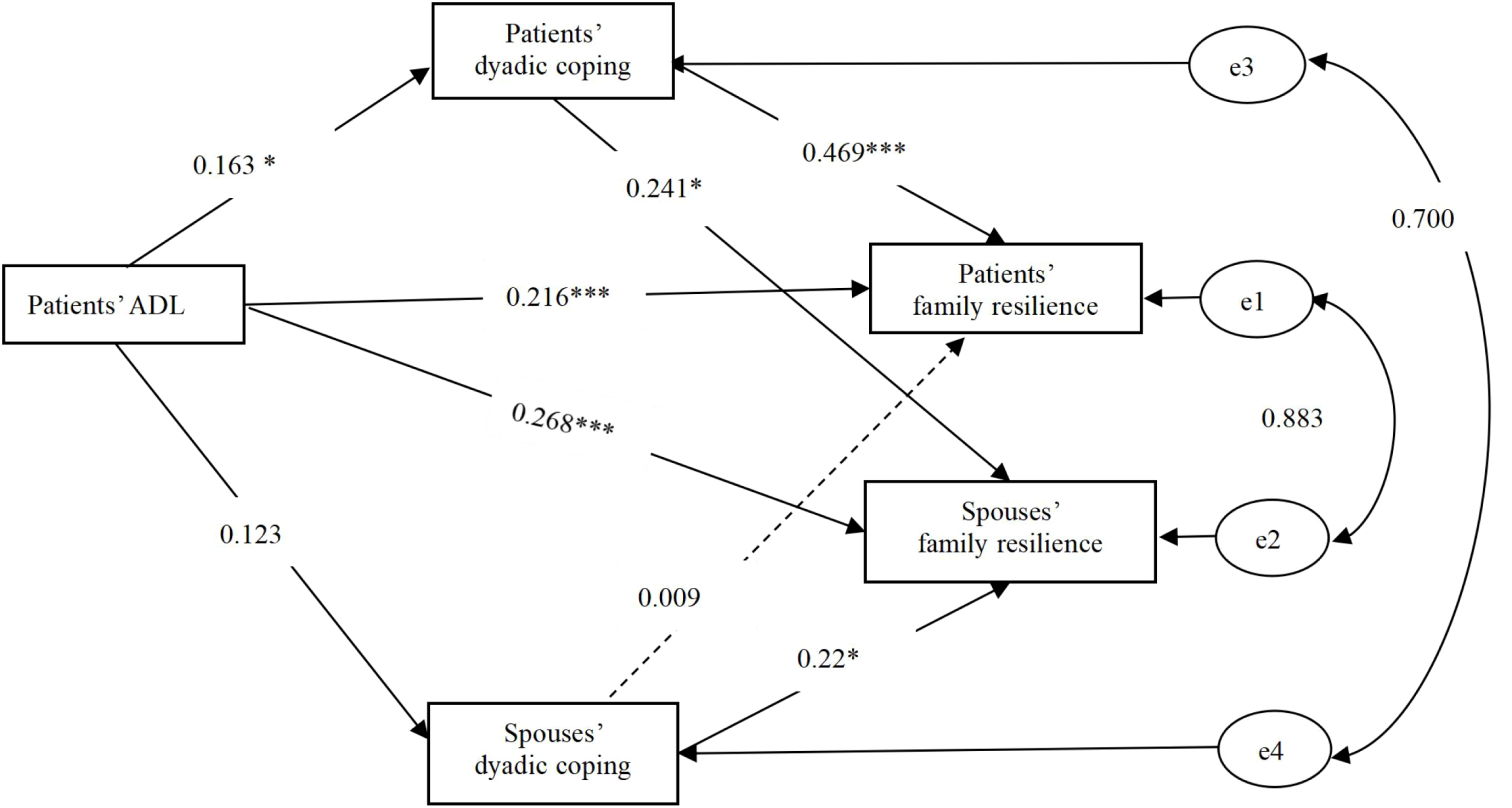
APIMeM results of ADL and dyadic coping on family resilience. In **Figure 4**, dotted lines represent non-significant path coefficients, while solid lines indicate statistically significant path coefficients. All path coefficients are standardized. Demographic variables were controlled for in the analysis. *P < 0.05, ***P < 0.001.

Our results showed that both patients’ and spouses’ depression had a statistically significant actor effect on their own family resilience (*β* = -0.718, *P* < 0.001). In contrast, their depression did not significantly affect their partners’ family resilience (*β* = 0.033, *P* = 0.820). Patients’ and spouses’ depression also had a significant actor effect on their own dyadic coping (*β* = -0.574, *P* < 0.001). In addition, significant partner effects were observed on the dyadic coping of partners (*β* = -0.450, *P* = 0.006). Furthermore, both patients’ and spouses’ dyadic coping had a significant positive effect on their own family resilience (*β* = 0.331, *P* < 0.001). Dyadic coping also positively influenced their partners’ family resilience (*β* = 0.125, *P* = 0.007). See **Figure 4** for details.

The mediation analysis showed that individuals’ dyadic coping partially mediated the relationship between their own depression and their family resilience (*β* = -0.190, 95%*CI* = -0.321~-0.084). Specifically, higher levels of dyadic coping can mitigate the negative effects of depression on family resilience, thereby fostering a more resilient family environment despite the presence of depression. Moreover, there was a significant actor-partner effect, indicating that better dyadic coping could also reduce the negative impact of a person’s depression on their partner’s family resilience (*β* = -0.072, 95%*CI* = -0.170~-0.017). See **Table 4** for further details.

**Table 4:** Total effects, indirect effects, and direct effects of patients’ and caregivers’ depression on family resilience via dyadic coping in the APIMeM.

		Unstandardized estimate	Lower	Upper	*P*
Actor effect (Individual’s anxiety →Individual’s FR)					
Total effects		-0.965	-1.316	-0.619	<0.001
Total IE		-0.247	-0.408	-0.113	0.001
**Actor-actor simple IE**	**Individual depression → individual DCI→ individual FR**	**-0.190**	**-0.321**	**-0.084**	**0.001**
Partner-partner simple IE	Individual depression → partner DCI→ individual FR	-0.056	-0.136	-0.013	0.006
**Direct effect**	**Individual anxiety→ individual FR**	**-0.718**	**-1.038**	**-0.397**	<0.001
Partner effect (Individual’s anxiety →Partner’s FR)					
Total effects		-0.188	-0.498	0.143	0.273
Total IE		-0.221	-0.381	-0.094	**<**0.001
**Actor-partner simple IE**	**Individual depression→ individual DCI→ partner FR**	**-0.072**	**-0.17**	**-0.017**	**0.006**
Partner- actor simple IE	Individual depression→ partner DCI→ partner FR	-0.149	-0.27	-0.054	0.002
**Direct effect**	**Individual depression → partner FR**	**0.033**	**-0.264**	**0.35**	**0.823**

IE, indirect effects; DCI, dyadic coping; FR, family resilience.

The bold values represent significant paths (P < 0.05, with the confidence interval not including 0).

#### 3.4.3 ADL, dyadic coping, and family resilience in stroke dyads

The hypothesized model (see **Figure 4**) provided a good fit to the data: *χ*²/df =1.815, RMSEA = 0.058, AGFI = 0.926, GFI = 0.997, NFI = 0.996, CFI = 0.998, TLI = 0.967. However, when *p* < 0.2 (*χ*²=3.63, *P* = 0.163), indicating a significant difference between the standardized and simplified models (22), we continued to use the standardized model to account for gender differences.

Our results showed that patients’ ADL had a statistically significant impact on their own family resilience (*β=*0.175, *P* < 0.001) and on their spouses’ family resilience (*β* = 0.198, *P* < 0.001). Additionally, patients’ ADL had a significant actor effect on their own dyadic coping (*β* = -0.833, *P* < 0.001). Finally, both patients’ and spouses’ dyadic coping had a statistically significant impact on their own family resilience (*β* = 0.492, *P* < 0.001). However, spouses’ dyadic coping did not significantly affect the patient’s family resilience (partner effect, *β* = 0.001,*P* = 0.993). See **Figure 4** for details.

In terms of indirect effects, as reported in **Table 5**, patients’ ADL was associated with their own family resilience through the partial mediation of dyadic coping (*β* = 0.062, 95%*CI* = 0.009 ~0.131) and was also associated with their spouses’ family resilience through the partial mediation of dyadic coping (*β* = 0.028, 95%*CI* = 0.002~0.079). See **Table 5** for details. (The *β* values in the results are unstandardized estimates).

**Table 5:** Total effects, indirect effects, and direct effects of patients’ ADL on family resilience via dyadic coping in the APIMeM.

Effect			Unstandardized estimate	Lower	Upper	*P*
Actor effect (Individual’s anxiety →Individual’s FR)						
patient	Total effects		0.237	0.116	0.362	0.001
	Total IE		0.062	0.008	0.118	0.026
	**Actor-actor simple IE**	**Patient ADL→ patient DCI→ patient FR**	**0.062**	**0.009**	**0.131**	**0.021**
	Partner-partner simple IE	Patient ADL→ spouse DCI→ patient FR	0.000	-0.025	0.028	0.927
	**Direct effect**	**Patient ADL→ patient FR**	**0.175**	**0.07**	**0.28**	**0.001**
Partner effect (Individual’s anxiety →Partner’s FR)						
spouse	Total effects		0.245	0.122	0.373	<0.001
	Total IE		0.048	0.002	0.095	0.039
	**Actor-partner simple IE**	**Patient ADL→ patient DCI→ spouse FR**	**0.028**	**0.002**	**0.079**	**0.027**
	Partner- actor simple IE	Patient ADL→ spouse DCI→ spouse FR	0.02	-0.001	0.068	0.062
	**Direct effect**	**Patient ADL→ spouse FR**	**0.198**	**0.089**	**0.306**	**0.001**

IE, indirect effects; DCI, dyadic coping; FR, family resilience.

The bold values represent significant paths (P < 0.05, with the confidence interval not including 0).

Finally, the results demonstrated that controlled demographic variables significantly influenced study outcomes in the path analyses (**Figures 2**–**4**). Patients with recurrent strokes exhibited lower levels of dyadic coping within couples compared to those experiencing their first stroke. Furthermore, individuals with mixed-type strokes showed reduced family resilience compared to those with hemorrhagic or ischemic strokes. Additionally, greater functional impairment in patients was associated with decreased family resilience for both patients and their spouses.

## 4 Discussion

This study provides fresh evidence on the dyadic mechanisms of family resilience in an understudied population—young stroke survivors and their spouses in China. While previous research has established the importance of dyadic coping in stroke (19), the specific role of dyadic coping in the family resilience of young stroke couples remains unclear. Moreover, our work specifically extends these findings by applying the APIMeM within a Chinese cultural framework, revealing how dyadic coping mediates the impact of both psychological and functional risk factors on family resilience in young dyads. All participating spouses confirmed they were the patient’s primary caregiver during the recovery period; therefore, the term “spouse” is used consistently throughout to refer to the caregiving partner.

First, our findings indicate that young stroke patients and their spouses exhibit family resilience levels that exceed the average. This corroborates (35), confirming that families affected by young-onset stroke can sustain or reestablish resilience despite enduring hardship. Nevertheless, our results also reveal that these patients experience more intricate outcomes than previously assumed, including profound physical and psychosocial repercussions (36). Numerous participants voiced apprehension about their future stability, indicating that persistent uncertainty and adversity erode family functioning and quality of life. Meanwhile, the majority of patients were aged 45–59 years, a life stage when individuals are at their peak of family and economic responsibilities, bearing the dual obligations of supporting elderly parents and raising children. Chinese culture emphasizes the inheritance of ethical values, with Confucian filial piety being the most prominent relational ethic. This entails children’s responsibility to support their parents, as well as the guardianship and upbringing of minor children. These duties and obligations are inherent challenges for young individuals. However, during illness, these responsibilities exacerbate patients’ concerns about the future (37). These insights underscore the urgent need for interventions that not only restore physical well-being but also fortify the family’s adaptive capacity early in the recovery process.

Second, consistent with prior studies (38, 39), we discovered that anxiety and depression in both patients and their spouses adversely influence their own family resilience. Elevated distress levels can undermine self-efficacy, engender feelings of entrapment, and constrain families’ capacity to seek external support. Spouses, as the primary caregivers, shoulder a dual burden: delivering long-term care and emotional support while fearing the loss of their partner. These psychological pressures directly impact family quality of life and long-term rehabilitation outcomes (40). During our study, a considerable proportion of families in our study had low monthly incomes and resided in rural villages—conditions that compound emotional distress through financial strain (41). The predominance of female spouses reflects traditional gender role expectations in Chinese society, where women serve as primary caregivers while often simultaneously managing household economic responsibilities. This dual burden intensifies the psychological toll on spouses, particularly when the male patient’s functional dependence increases. Furthermore, 37.04% of patients with recurrent strokes—a subgroup that exhibited significantly lower dyadic coping—face compounded psychological burden, consistent with the Double ABCX premise that cumulative stressors progressively deplete a family’s adaptive resources. Consequently, our findings highlight the necessity for early dyadic screening and mental health interventions to reduce anxiety and depression, particularly given the link between unmet care needs and emotional distress (42). The well-documented association between poverty and negative emotions, combined with the heightened financial stress observed in patients without stable incomes (43), makes workforce reintegration a critical component of family recovery—reducing financial strain can itself protect dyadic coping and resilience.

Notably, the anxiety and depression experienced by patients and their spouses did not significantly impact their partners’ family resilience. This implies that evaluating resilience from only one individual’s perspective may overlook the intricate dynamics of family systems. These results highlight the importance of a dyadic approach that recognizes both partners as contributors to and beneficiaries of family resilience.

Third, our findings indicate a strong association between patients’ ADL and family resilience: greater self-care capabilities correspond to enhanced resilience in both patients and their spouses. This aligns with previous research showing that severe physical symptoms can erode family resilience. As functional recovery advances, the family’s resilience also strengthens. Improved ADL not only alleviates caregiving burdens and resource constraints but also helps reestablish a dynamic equilibrium within the family (44). Integrating task-oriented ADL training into rehabilitation to ease caregiver burden (45), in tandem with psychosocial interventions, may therefore foster more comprehensive and enduring benefits.

A central and innovative aspect of this study lies in illustrating how dyadic coping mediates the influence of psychological and functional factors on family resilience. Although heightened anxiety diminishes dyadic coping, sustaining elevated levels of dyadic coping can offset these detrimental effects, ultimately reinforcing the family’s overall resilience. Moreover, dyadic coping not only mitigates the damaging impact of an individual’s depression on their own resilience, but also reduces the detrimental influence of one partner’s depression on the other’s resilience. These actor-partner effects emphasize that resilience does not arise in isolation; it is co-created within the couple, as each partner’s coping strategies shape both their own adaptive capacity and that of their partner. The stability and quality of a couple’s intimate relationship are crucial for alleviating the negative symptoms of illness (46, 47). In doing so, they help restore balance within the family.

Importantly, our findings also reveal that dyadic coping partially mediates the relationship between patients’ ADL and family resilience for both patients and spouses. Greater self-care abilities enhance dyadic coping, which in turn strengthens family resilience across the dyad. By integrating these functional, psychological, and relational dimensions within a single model, this study offers a more holistic understanding of how family resilience emerges from the complex interplay of individual traits, emotional well-being, and cooperative coping processes. These findings further suggest that improving family resilience requires reinforcing dyadic coping within young stroke dyads. Given current constraints in medical resources and geographic accessibility, we propose delivering interventions virtually. A video conferencing program centered on “Opening the Conversation” (48) can support survivors and spouses in improving communication, managing distress, and collectively coping with challenges, thereby enhancing the quality of couples’ coping strategies.

Finally, this study empirically verifies and extends the family resilience model in a Chinese cultural context, where collectivism is highly valued. McCubbin’s family resilience model posits that culture indirectly shapes the model’s core concepts by influencing cognitive systems—such as family structure, worldview, and paradigms (49). Giddens’ family structure theory similarly notes that differing cultural environments yield variations in resources, support, and interactional patterns (50). In Chinese culture, the family is regarded as the fundamental unit of society, and maintaining lineage and collective interests often surpasses individual priorities. Young adults play a pivotal role within this system; beyond supporting the elderly and raising children, they frequently bear economic and decision-making responsibilities. When a young adult experiences a sudden stroke, the repercussions for the family are profound: traditional roles are disrupted, and economic and emotional support systems must be reconfigured (37). The pronounced partner effects observed in our study diverge from patterns typically reported in Western contexts. Prior research on stroke dyads in individualistic cultures has reported significant actor effects of dyadic coping on quality of life, but the partner effects—particularly the mediation of partner effects through dyadic coping—appear less prominent (19). This divergence likely reflects a culturally conditioned preference for internal resource mobilization in Chinese families. Our clinical research indicates that following a patient’s illness, family members promptly allocate resources (e.g., assistance from relatives, savings, and social connections). Simultaneously, families’ perceptions and evaluations of the illness, along with their chosen coping strategies, exhibit distinctive patterns in the Chinese context. For instance, mutual support between partners involves not merely emotional solace but also the redistribution of responsibilities in decision-making, daily care, and childrearing. In Western cultures, where professional psychosocial services are more commonly integrated into stroke care pathways and personal autonomy is emphasized (51), some of the adaptive burden carried by dyadic coping in our sample may be absorbed by external support systems. The traditional gender roles evident in our sample—69.55% female spouses as primary caregivers—further shape these dynamics. amplifying the partner effects of patients’ functional dependence on spouses’ resilience through the culturally expected caregiving pathway. Consequently, couple-based interventions that strengthen dyadic coping are not only effective but culturally congruent: they work with, rather than against, the family’s natural inclination to handle adversity through internal resource mobilization, strengthening the very coping mechanisms that Chinese families already preferentially employ.

Using evidence-based methods, this study identifies and validates anxiety, depression, and the self-care abilities of both stroke survivors and their spouses as key family risk factors. Family resilience varies meaningfully among young stroke couples, highlighting the need for future research to account for this diversity. The findings further suggest that couples can mitigate the impact of these risk factors through active coping strategies, improved marital quality, and open communication, thereby facilitating mutual recovery from adversity. By grounding these insights within the context of Chinese collectivist values, this study deepens and expands the family resilience model for young stroke patients.

Several limitations of this study should be considered. First, the cross-sectional nature of this study precludes establishing causal relationships; thus, future longitudinal research is recommended to clarify directional influences. Second, recruitment from four hospitals in a single Chinese province, although our sample size was adequate for current analyses and yielded valuable insights into how family resilience is shaped by risk factors and dyadic coping, a larger sample may improve generalizability. Third, all participating spouses were the primary caregivers, yet we did not assess relationship quality, duration, or broader social support networks beyond the dyad, all of which could influence dyadic coping effectiveness. Fourth, socioeconomic data were limited to income; other indicators such as education or occupation were not fully integrated into the model. Notably, the majority of participants had completed junior high school education or below (68.31% of patients, 77.37% of spouses), which may have influenced their understanding of questionnaire items and their health literacy in managing stroke-related challenges. Fifth, due to patients’ conditions, we were unable to separate patients and spouses for extended periods during questionnaire administration, raising the possibility of some mutual influence in their responses despite our efforts to ensure independent completion. Finally, the study did not account for the potential roles of other family members, which may be particularly relevant in Chinese extended family structures where intergenerational support is common.

## 5 Conclusions

This study demonstrates that dyadic coping serves as a key mediator linking family risk factors—anxiety, depression, and ADL—to family resilience in young stroke dyads. The APIMeM findings reveal that resilience is not an individual attribute but a shared phenomenon co-constructed through both partners’ collaborative coping behaviors. This dyadic interdependence is particularly pronounced within the Chinese cultural context. Accordingly, rehabilitation for young stroke survivors should be conceptualized as a couple-based intervention in which emotion regulation and collaborative coping skills are targeted simultaneously to strengthen the family’s adaptive capacity.

## Data Availability

The original contributions presented in the study are included in the article/**Supplementary Material**. Further inquiries can be directed to the corresponding authors.
