# Supplementary material for: Family risk factors, dyadic coping, and family resilience in young stroke dyads: an actor-partner interdependence mediation model
Source: Front Psychiatry. 2026 Jun 5;17:1826436. doi: 10.3389/fpsyt.2026.1826436 (PMC13279724; doi:10.3389/fpsyt.2026.1826436)
Supplement: Supplementary file 1 [file SupplementaryFile1.docx]

| **Table Effects of different patient characteristics on family resilience of stroke patients and their spouses**  **（Ｎ＝243）** | | | | | |
| --- | --- | --- | --- | --- | --- |
| **Characteristic/variables** | | **Stroke survivors** | ***t/F*** | **Spouses** | ***t/F*** |
|  |  | **Means (SD) or n (%)** |  | **Means (SD) or n (%)** |  |
| Sociodemographic information | |  |  |  |  |
| Gender | Man | 3.68±0.39 | 0.611 | 3.71±0.33 | 0.922 |
|  | Woman | 3.65±0.39 |  | 3.67±0.41 |  |
| Age | ＜45 | 3.63±0.368 | -0.488 | 3.71±0.32 | 0.112 |
|  | 45-59 | 3.67±0.395 |  | 3.70±0.36 |  |
| Educational background | Primary school and below | 3.61±0.37 | 0.997 | 3.63±0.38 | 1.942 |
|  | Junior high school | 3.67±0.43 |  | 3.71±0.38 |  |
|  | High school or polytechnic school | 3.68±0.39 |  | 3.69±0.30 |  |
|  | Junior college | 3.83±0.27 |  | 3.89±0.30 |  |
|  | Bachelor degree or above | 3.79±0.11 |  | 3.87±0.18 |  |
| Occupational status | Employed | 3.67±0.36 | 2.365 | 3.68±0.35 | 1.313 |
|  | Self-employed | 3.69±0.39 |  | 3.72±0.35 |  |
|  | Unemployed | 3.42±0.53 |  | 3.54±0.36 |  |
| Clinical information |  |  |  |  |  |
| Type of stroke | Ischaemic stroke | 3.72±0.35 | 13.119** | 3.74±0.29 | 12.516** |
|  | Haemorrhagic stroke | 3.32±0.53 |  | 3.40±0.59 |  |
|  | Mixed type | 3.66±0.40 |  | 3.60±0.43 |  |
| Comorbid states | 0-1 | 3.70±0.37 | 2.117 | 3.74±0.36 | 1.492 |
|  | 2-3 | 3.68±0.39 |  | 3.69±0.36 |  |
|  | 4-6 | 3.52±0.49 |  | 3.60±0.31 |  |
| First stroke | Yes | 3.72±0.36 | 2.461* | 3.74±0.34 | 2.274* |
|  | No | 3.59±0.44 |  | 3.63±0.38 |  |
| Dysfunction | 0-1 | 3.79±0.36 | 14.668** | 3.79±0.29 | 11.641** |
|  | 2-3 | 3.57±0.38 |  | 3.63±0.38 |  |
|  | 4-6 | 3.29±0.48 |  | 3.33±0.47 |  |
| Family information |  |  |  |  |  |
| Monthly per capita income (CNY) | ＜ 3000 | 3.66±0.41 | 0.378 | 3.70±0.38 | 0.794 |
|  | 3000-5000 | 3.70±0.34 |  | 3.72±0.30 |  |
|  | ＞5000 | 3.57±0.51 |  | 3.60±0.50 |  |
| Permanent home address | Village | 3.71±0.39 | 0.840 | 3.72±0.36 | 0.403 |
|  | Counties | 3.65±0.39 |  | 3.66±0.40 |  |
|  | City | 3.64±0.40 |  | 3.69±0.34 |  |
| **P*<0.05;***P*<0.05 | | | | | |

**Supplementary file 1. Effects of Stroke Patients' Characteristics on Family Resilience of Both Patients and Their Spouses**
