# Supplementary material for: Family risk factors, dyadic coping, and family resilience in young stroke dyads: an actor-partner interdependence mediation model
Source: Front Psychiatry. 2026 Jun 5;17:1826436. doi: 10.3389/fpsyt.2026.1826436 (PMC13279724; doi:10.3389/fpsyt.2026.1826436)
Supplement: Supplementary file 2 [file SupplementaryFile2.docx]

| **Table Current Status of Scores for variables（Ｎ＝243）** | | | | | |
| --- | --- | --- | --- | --- | --- |
| **Variables** | **Stroke survivors** |  | **Spouses** | ***t*** | ***P*** |
|  | **‾*x±s*** |  | ***‾x±s*** |  |  |
| Family resilience | 3.67 ±0.39 |  | 3.70 ±0.36 | -1.978 | 0.049 |
| Dyadic coping | 3.23±0.52 |  | 3.26±0.52 | -1.725 | 0.086 |
| Anxiety | 1.56±0.66 |  | 1.34±0.53 | 6.169 | ＜0.001 |
| Depression | 1.56±0.72 |  | 1.31±0.53 | 6.505 | ＜0.001 |

**Supplementary file 2. Current Status of the Scores for Stroke Patients and Their Spouses' Variables**
